# *Melanocortin receptor 1* and black pigmentation in the Japanese ornamental carp (*Cyprinus carpio* var. Koi)

**DOI:** 10.3389/fgene.2013.00006

**Published:** 2013-01-25

**Authors:** Ido Bar, Ethan Kaddar, Ariel Velan, Lior David

**Affiliations:** Department of Animal Sciences, R. H. Smith Faculty of Agriculture, Food and Environment, The Hebrew University of JerusalemRehovot, Israel

**Keywords:** pigmentation development, melanophore, larva, fish, color, mc1r

## Abstract

Colors and their patterns are fascinating phenotypes with great importance for fitness under natural conditions. For this reason and because pigmentation is associated with diseases, much research was devoted to study the genetics of pigmentation in animals. Considerable contribution to our understanding of color phenotypes was made by studies in domesticated animals that exhibit dazzling variation in color traits. Koi strains, the ornamental variants of the common carp, are a striking example for color variability that was selected by man during a very short period on an evolutionary timescale. Among several pigmentation genes, genetic variation in *Melanocrtin receptor 1* was repeatedly associated with dark pigmentation phenotypes in numerous animals. In this study, we cloned *Melanocrtin receptor 1* from the common carp. We found that alleles of the gene were not associated with the development of black color in Koi. However, the mRNA expression levels of the gene were higher during dark pigmentation development in larvae and in dark pigmented tissues of adult fish, suggesting that variation in the regulation of the gene is associated with black color in Koi. These regulatory differences are reflected in both the timing of the dark-pigmentation development and the different mode of inheritance of the two black patterns associated with them. Identifying the genetic basis of color and color patterns in Koi will promote the production of this valuable ornamental fish. Furthermore, given the rich variety of colors and patterns, Koi serves as a good model to unravel pigmentation genes and their phenotypic effects and by that to improve our understanding of the genetic basis of colors also in natural populations.

## Introduction

### Mc1r and dark pigmentation in animals

In natural populations, color variation has multiple adaptive roles related to intraspecific communication, interspecific interactions, photoprotection, photoreception, and thermoregulation (Hubbard et al., 2010). In domestic animals, however, most color phenotypes were selected due to their attractiveness to the eye of the beholder and not due to their adaptive roles. Furthermore, unlike the slower rate of phenotypic changes in natural populations, the myriad color phenotypes specific to domestic animals were developed during a relatively short evolutionary time following their domestication (Cieslak et al., 2011).

Unraveling the genetic variation underlying color traits variation has improved our understanding of pigmentation biology and the evolution of visual communication. DNA sequence variations in several genes were associated with differences in color phenotypes in animals (Mundy, 2005; Mills and Patterson, 2009; Hubbard et al., 2010). Similar coat color phenotypes between natural and domesticated species were often a result of mutations in the same genes among which, most notably, were mutations in the *Melanocortin receptor 1 gene* (*mc1r*). *mc1r* is one member in a family of five Melanocortin receptor (*mcr*) genes found in vertebrates. This gene codes for a seven trans-membrane (TA) domains G-Protein Coupled Receptor (GPCR), which mediates the physiological action of melanocortins by a G-protein-dependent activation of a cyclic AMP (cAMP) signaling pathway (Ito, 2003; Garcia-Borron et al., 2005). *mc1r* which is expressed in melanocytes of mammals and birds, regulates the amount and type of melanin production. Higher activity of the pathway leads to production of eumelanin and black/brown pigmentation whereas lower activity to production of pheomelanin and red/yellow pigmentation (Ito, 2003; Garcia-Borron et al., 2005; Metz et al., 2006). Often, *mc1r* mutations were found to have a partial to full dominant effect resulting in black color of fur, skin, or plumage (Vage et al., 1999; Kijas et al., 2001; Andersson, 2003; Mundy, 2005; Hoekstra, 2006; Beaumont et al., 2007).

Whereas in mammals and birds, one type of pigment cells was found (the melanocyte), up to six types, known as chromatophores, were described in fish (Kelsh, 2004). In addition to genetically inherited changes in coat color, fish also display rapid physiological changes in coloration in response to environmental changes. Such physiological changes do not rely on mutations but rather on regulation of local concentrations of melanin and melanophores as well as of other chromatophores in different areas of the skin. This response is also dependent on induction of the cAMP signaling pathway by *Mc1r* and therefore subjected to genetic variation in its sequence and regulation (van der Salm et al., 2005; Logan et al., 2006; Richardson et al., 2008).

### The black color of Koi

The Japanese ornamental carp, Koi, is the colorful variant of the common carp (*Cyprinus carpio* L.). The common carp became the first domesticated fish about 2000 years ago and since then it is produced as a major food fish species in Eastern Europe and South Asia. Colored variants of the carp were isolated and cultured in China for many centuries (Balon, 1995). However, selection of the colored and patterned Koi started only a couple of centuries ago in Japan and due to intensive selection and crossbreeding, it took only a few decades to isolate the many Koi varieties existing today. In the last couple of decades, Koi became an appreciated and expensive pet in Europe and North America as well (Axelrod, 1988; Balon, 2004). The quick establishment of variable colors and color patterns under domestication makes Koi a unique system for studying the genetics of pigmentation. Identifying the genetic basis of colors and color patterns in Koi will promote breeding of this fish and will enhance our understanding of pigmentation biology and development.

Among the varieties of Koi, two major black color patterns were defined: the Bekko and Utsuri patterns, which are distinguished by small black dots and large patches of black, respectively. Both patterns can be found along with red, white, or both and each of these combinations is considered a different variant by the producers and hobbyists (Axelrod, 1988). Previous studies started addressing the mode of inheritance and the development of the Bekko and Utsuri pattern. The initial development of both patterns involves the formation of black melanophores on the skin of larvae. Melanophores that will lead to the Utsuri pattern in fry, here termed dark-early, can be seen on the body of the larvae already at hatching (48–72 h post-fertilization), while those that will lead to the Bekko pattern, here termed dark-late, appear in larvae only around 2 weeks post hatching (David et al., 2004). Besides the difference in developmental times, the mode of inheritance of these two patterns is different. The Bekko pattern is most likely controlled by a single gene with a dominant black allele while the Utsuri pattern is probably multigenic (Gomelsky et al., 1998; David et al., 2004).

The association of *mc1r* mutations with dark coat color in so many animals (including fish) and the typical monogenic dominant effect of these mutations, make *mc1r* a natural candidate for controlling black color and pattern in Koi. To test this hypothesis, here we isolated the gene sequence from carp and analyzed the linkage between its alleles and the Bekko pattern in Koi families. We also characterized the mRNA expression of this gene in adult fish and during development of dark pigmentation.

## Materials and methods

### Fish families production and analysis

Koi fish from the studied varieties were sampled by random from the Gan-Shmuel Fish Breeding Center, Kibbutz Gan-Shmuel, Israel. Koi varieties included Ohgon (platinum white), Kohaku (red patches over white body), Sanke (red patches and Bekko black spots over white body), and Utsuri (Black patches over white body). Common carp strains included the wild/feral Sassan strain, the Dor-70 and Yugoslavian (Našice) strains (Shapira et al., 2005). These fish were used for *mc1r* genotype analysis. Parent Koi fish, which were used for production of the experimental families, were induced to spawn by hormone injection according to standard protocols (Yaron et al., 2009). Eggs and sperm were mixed manually according to the crossing scheme and fertilized eggs of each family were incubated separately in running fresh water at a temperature of 24°C. After hatching, groups of a few thousand larvae were kept separately in conical tanks of 60 liter with running water and aeration. Larvae were fed with live *Artemia* in the first couple of weeks that was gradually replaced by fine dry food according to size. For the crosses involving Utsuri parents, the larvae were sorted and counted at day 1 post hatching and the dark-early and light groups were kept in separate tanks. For later time points, the dark-late/light ratio was calculated in counted random samples from the originally all-light group and besides the few larvae that were sampled for DNA or RNA analysis, the larvae were returned to their original tanks. In addition, subset groups of dark-late from these two crosses were kept separately for easier sampling. The larvae from families of Bekko parents were treated like the light larvae group described earlier. As the larvae grew, to reduce the density and promote growth, the groups were culled randomly until about 1000 fry from each family were moved to 500 liters tanks for further growth. Fish reproduction, rearing, sampling and dissection were carried out using appropriate procedures with accordance to and permission from the animal research ethics committee of the Hebrew University of Jerusalem.

### DNA extraction

Caudal fin clips were taken from the fish and stored in 100% Ethanol at −20°C. DNA was extracted using modified protein salting-out method (Martinez et al., 1998). In brief, 5 mg of a fin sample was dried from ethanol and placed in 550 μL of cell lysis solution (50 mM Tris- HCl, pH 8.0, 50 mM EDTA, 100 mM NaCl). SDS solution was added to a concentration of 1% and cells were further lysed by incubation with 1 μL Proteinase K solution (20 mg/mL) for 2 h at 50°C. The lysate was supplemented with 300 μL of 5 M NaCl, vortexed and centrifuged (13,000 rpm, 10 min) to precipitate the proteins. The supernatant liquid phase was mixed with 900 μL of freezer cold 100% isopropanol, incubated for 2 h at −20°C and centrifuged (13,000 rpm, 5 min). The resulting DNA pellet was washed with 700 μL of 70% ethanol, dried for 15 min and dissolved overnight in 100 μL of double distilled water at 4°C. DNA concentration and quality (Optical Density OD_260_/OD_280_ ratio) were measured using NanoDrop ND-1000 (NanoDrop Technologies) and examined by 1.5% TBE Agarose gel electrophoresis. DNA samples were diluted to a concentration of 25 ng/μL and stored at −20°C for further analysis.

### RNA extraction and cDNA synthesis

Adult fish were anesthetized with 2-phenoxyethanol and sacrificed. Tissue samples or whole larvae were frozen immediately in liquid nitrogen and stored at −80°C for later use. Pools of 5–15 individuals of the same phenotype were used to obtain sufficient RNA for analysis of early stages larvae. Samples were placed on ice to thaw in 1 mL of TRIzol® Reagent (Invitrogen) and then homogenized using a T-25 (IKA systems) homogenizer (13,000 rpm, 1 min). The homogenate was centrifuged (11,000 rpm, 10 min at 4°C) for protein precipitation. The supernatant was mixed with 200 μL of chloroform and centrifuged again (11,000 rpm, 15 min at 4°C). The aqueous phase (~400 μL) were transferred to a new tube, mixed with 500 μL of isopropanol, incubated overnight at −20°C and then centrifuged (11,000 rpm, 10 min at 4°C). The resulting RNA pellet was washed twice with 500 μL of 75% ethanol, air dried on ice for 30 min and dissolved in 20 μL of diethyl dicarbonate (DEPC) double distilled water for 30 min at 4°C. RNA concentration and quality (Optical Density OD_260_/OD_280_) were measured using NanoDrop ND-1000 (NanoDrop Technologies). One microgram of RNA was analyzed for integrity by electrophoresis on 1.5% TBE-agarose gel stained with ethidium bromide. RNA samples were diluted to a concentration of 1 μg/μL, treated with Turbo DNase (Ambion) according to the manufacturer's protocol and stored at −80°C for further analysis. Representative samples from each batch of RNA extraction were analyzed for quality using the Bioanalyzer (Agilent technologies).

cDNA was synthesized using M-MLV Reverse Transcriptase (Promega) according to the manufacturer's protocol. In brief: 2 μg DNase treated RNA, dissolved in 15 μL DEPC double distilled water, were denatured with 1 μL of OligodT_15−18_ primer (0.5 μg/μL) for 5 min at 70°C and cooled down to 4°C. Nine microliters of reaction mix containing dNTPs, ×5 buffer, Bovine Serum Albumin, RNASin and Reverse Transcriptase, were added to each sample to a final reaction volume of 25 μl. The same reaction mix, without Reverse Transcriptase (RT-) was used as negative control for each sample. cDNA synthesis was conducted in the thermocycler at 42°C for 60 min followed by 10°C for 2 min. One microliter of the cDNA product and the negative control (RT-) were used to verify cDNA synthesis and absence of genomic DNA contamination by PCR amplification across an intron of the *b-actin* gene (Table 1) yielding a 210 bp product for cDNA template, or 303 bp product for genomic DNA template. PCR products were analyzed by electrophoresis on 1.5% TBE agarose gel.

**Table 1:** **List of primers and their use in the study**.

**Name**	**Sequence 5′-3′**	**Used for**	**Remarks**
MC1Rgsp_Ex5′RACE_R	TGCGCAGCTCCTGACTGCGATAC	5′ transcript cloning	External paired with the universal 5′ RACE primer
MC1Rgsp_Ex3′RACE_F	GGTTGCGGCCATCATCAAGAACA	3′ transcript cloning	External paired with the universal 3′ RACE primer
MC1Rgsp_In5′RACE_R	GGATGAGGATGAGGTGGAGA	5′ transcript cloning	Internal (nested) paired with the universal 5′ RACE primer
MC1Rgsp_In3′RACE_F	ATGCAGCTCTGTCGTTTCCT	3′ transcript cloning	Internal (nested) paired with the universal 3′ RACE primer
1_MC1R, f	CGGGAGAGGGAAAAGAGAT	*mc1r* sequencing	
1_MC1R, r	CTTCAGCCCACCTTTGACAT	*mc1r* sequencing	
2_MC1R, f	AAAGGTAAAACTGCTTGTGTTCG	*mc1r* sequencing	
2_MC1R, r	AGGAAACGACAGAGCTGCAT	*mc1r* sequencing	
3_MC1R, f	ATGCTGGTGAGCGTCAGTAA	*mc1r* sequencing	
3_MC1R, r	GGATGAGGATGAG GTGGAGA	*mc1r* sequencing	
4_MC1R, f	GGGAGCCATTACTCTGACCA	*mc1r* sequencing	
4_MC1R, r	CATGTCATATGCTGAGCCACA	*mc1r* sequencing	
MC1R_SSR_FL_F	GTTTTCCCAGTCACGACAAAAGGCGCTCCTATGATT	*mc1r* microsatellite	
MC1R_SSR_FL_R	GTTTGCAATTGCGTTTTCGAGATT	*mc1r* microsatellite	
5′ fluorescent-labeled	GTTTTCCCAGTCACGAC	*mc1r* microsatellite	FAM, PET, NED or VIC labeled
*b_ACTIN* exon4-6_f	AGGTGCCCAGAGGCCCTGTT	cDNA contamination analysis	Across exon, yielding different size for cDNA and gDNA
*b_ACTIN* exon4-6_r	CATTGTGCTGGGGGCCAGGG	cDNA contamination analysis	Across exon, yielding different size for cDNA and gDNA
*EF1α*_f	CAAGGTCACGAAGTCTGCAC	mRNA, control	
*EF1α*_r	CACGAGGTTGGGAAGAACAT	mRNA, control	Product size 98 bp
18s ribosomal RNA_f	AAACGGCTACCACATCCAAG	mRNA, control	
18s ribosomal RNA_r	TTACAGGGCCTCGAAAGAGA	mRNA, control	Product size 110 bp
*b_ACTIN*_f	ACTGCTGCTTCCTCCTCCTC	mRNA, control	
*b_ACTIN*_r	CATTGTGCTGGGGGCCAGGG	mRNA, control	Product size 88 bp
MC1R_Short_f	CATTCTTGCAAATAGCGTCCT	mRNA levels	
MC1R_Short_r	TCAGTCAAAGAGTTTTGCGTCT	mRNA levels	Product size 85 bp

### Cloning of *mc1r* from the common carp

An alignment of *mc1r* sequences of various fish species was used for identifying the conserved regions of the gene. A pair of primers in conserved regions was designed based on the sequence of the zebrafish gene. These primers gave the expected size product from genomic DNA of the common carp and sequencing confirmed the common carp *mc1r* (*ccmc1r*) specific amplification. For cloning of the full gene, RNA was extracted from the dark skin of the common carp. Full cDNA sequence of *mc1r* was isolated by 5′ and 3′ RACE (Rapid amplification of cDNA ends) using the RACE kit of Clontech Inc. and according to the manufacturer protocol. Based on the partial *ccmc1r* sequence, two pairs (external and internal) of 5′ and 3′ gene specific primers were designed (Table 1). First round of 5′ and 3′ RACE was done using the external primers and a second round using the internal (nested) ones. The products of the second round were sequenced and the sequences were aligned to determine the full transcript sequence of the gene.

### Gene sequencing and microsatellite genotyping

Based on the cDNA sequence obtained, *mc1r* was further amplified from genomic DNA using four primer pairs covering overlapping regions of the entire *mc1r* transcript sequence (Table 1). The PCR profile used for amplification was 94°C for 10 min, followed by a touchdown profile: 94°C for 30 s, 60–53°C for 1 min with a decrease of 0.5°C per each of 14 cycles and extension at 72°C for 2 min. The touch-down was followed by 20 cycles with annealing temperature of 53°C and a final elongation step of 72°C for 10 min. PCR products were verified by electrophoresis on 1.5% TBE agarose gel, cleaned using ExoSAP-IT (USB, Cleveland, OH) and sequenced using big dye chemistry on an ABI PRISM 3730xl DNA Analyzer (The Center for Genomic Technologies, Hebrew University, Jerusalem).

Genotyping of the *mc1r* microsatellite was carried out by PCR on genomic DNA of individuals using two marker specific primers and a third primer without homology to the carp genome, 5′ labeled by a fluorescent dye (Table 1). The forward marker-specific primer contained a 5′ tail homologous to the third fluorescent primer sequence. Four primers with identical sequence but each labeled with a different fluorescent dye allowed pooling of PCR products from four individuals. Fragment size separation was done on an ABI PRISM 3730xl DNA Analyzer and the fragment sizes were determined using the GeneMapper Software (Applied Biosystems).

### *Mc1r* comparative protein sequence analysis

Phylogeny was reconstructed from 17 *Mc1r* protein sequences (NCBI accession number follows each species name) from 13 fish species (*Cyprinus carpio*, JX989223; *Danio rerio*, NP_851301.1; *Oreochromis mossambicus*, CAI38756.2; *Oncorhynchus mykiss*, NP_001182107.1; *Psetta maxima*, ACN38801.1; *Poecilia reticulate*, BAJ72964.1; *Astyanax mexicanus*, ACN39571.1; *Takifugu rubripes*, AAO65548.1; *Xiphophorus maculates*, ABI34468.1; *Tetraodon nigroviridis*, AAQ55176.1; *Dicentrarchus labrax*, CAY39344.1; *Paralichthys olivaceus*, ABY77479.1; *Nothobranchius furzeri*, ADB54831.1), one amphibian (*Rana temporaria*, ACA28876.1), chicken (*Gallus gallus*, NP_001026633.1) and two mammals (*Mus musculus*, NP_032585.2 and human: *Homo sapiens*, NP_002377.4). Two other members of the *Mcr* gene family from common carp were included in the analysis: *Mc2r* (CAE53845.1) and *Mc5r* (CAH04350.1). The phylogeny was inferred by calculating pairwise genetic distances and clustering using the Neighbor-Joining method (Saitou and Nei, 1987), as implemented in MEGA4 (Tamura et al., 2007). The statistical significance of the phylogeny was inferred by the bootstrapping method (1000 replicates) and a consensus tree was inferred.

### Protein structure prediction

TM helices and topology of the Mc1r protein were predicted based on its amino acid sequence, using HMMTOP automatic server [http://www.enzim.hu/hmmtop/; (Tusnady and Simon, 2001)]. Post-translational phosphorylation and glycosylation sites were predicted using online prediction servers [http://www.cbs.dtu.dk/services/; (Blom et al., 2004)]. *ccMc1r* amino acid sequence was plotted according to prediction, and for conservation analysis compared to those from thirteen fish species: *D. rerio, A. mexicanus, N. furzeri, T. chinensis, T. rubripes, T. nigroviridis, X. maculates, O. mossambicus, V. moseri* (accession number AB287974), *O. mykiss, P. maxima, D. labrax, P. reticulata*. Topology plot was drawn with T(E)Xtopo package, using “Similarity” shading mode, with a threshold of 50% (Beitz, 2000).

### RT-qPCR

Relative expression of *mc1r* was measured by real-time RT-qPCR. Primers for elongation factor 1-alpha (*ef1a*), *18s* ribosomal RNA and *b-actin* reference genes were designed based on *C. carpio* EST libraries from NCBI. Primers for measuring the expression levels of *mc1r* were designed based on the cloned transcript (Table 1). Calibration curves were generated for each gene to calculate amplification efficiency and the preferred amount of cDNA template to use. To generate a calibration curve, a pool of cDNA samples was created and diluted to the concentrations of 3 μg/μL, 750 ng/μL, 188 ng/μL, and 47 ng/μL for *mc1r*, and 600 ng/μL, 120 ng/μL, 24 ng/μL, and 4.8 ng/μL for reference genes. RT-qPCR analysis was carried out on an Mx3000P thermo cycler (Agilent Technologies) with the following reaction master mix: 5 μL water, 2 μL of primer mix (forward + reverse, 2 μM) and 10 μL Platinum SYBR Green qPCR SuperMix-UDG (Invitrogen). Seventeen milliliters of the master mix were placed into each well of a Thermo-Fast 96 detection plate (Thermo Scientific) and complemented by 3 μL of the cDNA sample (1.8 μg for *mc1r* target gene and 60 ng for *ef1a, 18s*, and *b-actin* reference genes) as PCR template. The plate was covered with ABsolute qPCR Optically Clear Adhesive Seal (Thermo Scientific), centrifuged and placed in the Mx3000P thermo cycler. The following Mx3000P experimental protocol was used: pre-amplification hot start segment (50°C for 2 min, 95°C for 2 min), amplification segment (95°C for 30 s, 62°C for 1 min with a single fluorescence measurement), repeated for 40 cycles and a dissociation curve segment (95°C for 1 min, 55°C for 30 s and ramp up to 95°C, with increment of 0.01°C per second and continuous fluorescence measurement). Ct values were measured at fluorescence threshold of 500 dR. Thermo cycler fluorescence results were analyzed with MxPro v4.1 software (Stratagene). After calibration of all primers, *ef1a* was chosen as the reference genes for further analyses of *mc1r* expression levels.

### Data analysis

Relative expression of *mc1r* was calculated using the “−ΔΔCp with efficiency correction” calculation method (Pfaffl, 2001). In this method, levels of the query gene expression are normalized first relative to a reference gene that is common to all samples. Then, the normalized expression levels of the query gene in the different samples are analyzed relative to its expression in a reference sample. Here, the expression level of *mc1r* in larvae was normalized to *ef1a* and calculated relative to its expression in light larvae at day 14. Common samples were added to each plate in order to normalize between experiments in different days and plates. Expression in larvae of each pigmentation group and day was measured in 3–15 replicates. Tissue expression levels in adult carp were measured relative to *ef1a*, using white skin as control tissue. Three to five biological replicates were taken from each tissue. Expression ratio values were transformed (Fold change^0.25) to obtain normally distributed values and equal variances. Differences in expression ratios between days in each phenotypic group were evaluated by Tukey HSD test or by Student's *T*-test. Goodness of fit between the observed and expected ratios of genotypic segregation of *mc1r* alleles was evaluated by a χ^2^ test. Statistical analyses for all parts of the results were done using the JMP8 software (SAS institute, NC).

## Results

### *Cyprinus carpio mc1r* sequence

**Figure 1:**
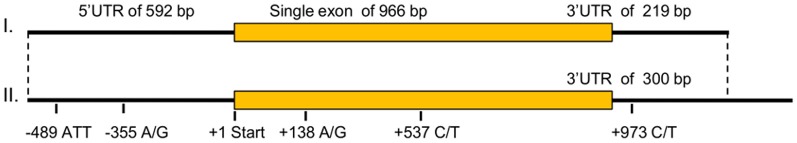
**The *ccmc1r* genomic structure, transcripts, and polymorphisms.** *ccmc1r* is a single exon gene and thus, its genomic and transcript structures are identical. Shown are the size (in bp) of the 5′UTR, ORF and 3′UTR of the gene. Two transcripts were identified that differ only in the size of their 3′UTR. The position of polymorphisms relative to the translation start site is given below. Note that the −489 polymorphism is a microsatellite with an ATT motif while all other four are SNPs.

**Figure 2:**
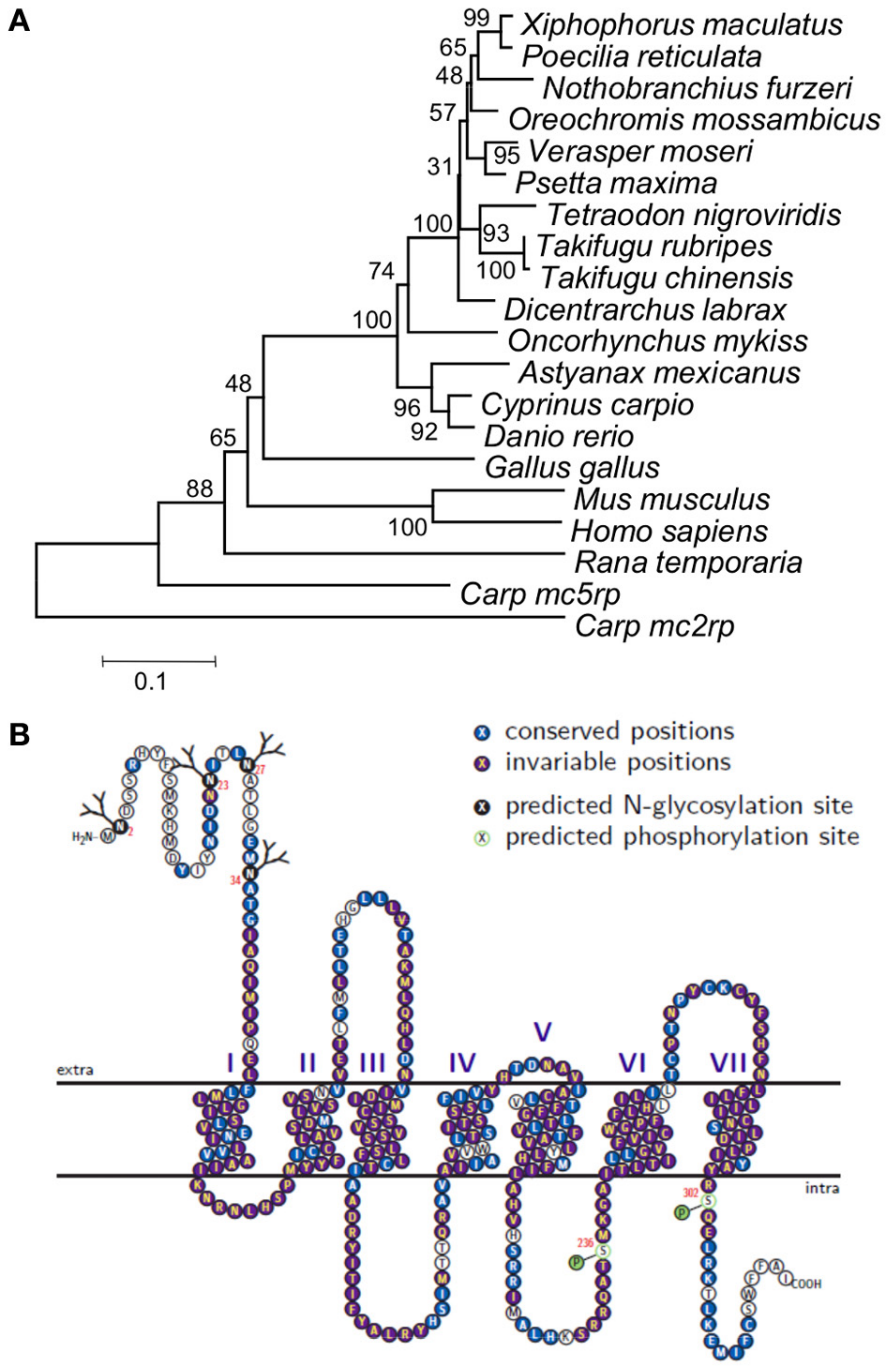
**Phylogeny, topology, and evolutionary conservation of *ccMc1rp*. (A)** Phylogeny based on alignment of *Mc1r* from 14 fish species, chicken, frog, mouse, and human. Sequences of *Mc2rp* and *Mc5rp* of carp were also included. Phylogeny was built using the Neighbor-joining method and numbers next to nodes are % support based on 1000 bootstraps. **(B)** The predicted *ccMc1rp* topology including the seven trans-membrane domains as well as intra- and extra-cellular loops and tails. Each residue is marked by a circle and the colors denote glycosylation and phosphorylation sites as well as conservation levels based on the alignment of the 14 fish species.

The common carp *mc1r* DNA sequence was unknown prior to this study. Cloning of the gene transcripts from skin cDNA revealed two transcripts (Figure 1), differing in size by 81 bp (GenBank accession no. JX989223). Since one had a shorter 3′ UTR than the other did, this gene has alternative transcription stop sites. Both transcripts contain a 966 bp long open reading frame (ORF), which translates to a 321 amino acids long protein. Further sequencing of the gene from genomic DNA confirmed that like in other fish species, the common carp *mc1r* gene structure includes a single exon (Figure 1). Comparison of *ccmc1r* DNA and amino acid sequences to these of another cyprinid, the zebrafish (*D. rerio*), revealed 87% and 93% homology, respectively. The clustering of *ccMc1rp* with Mc1r proteins from other species and separately from other Mcr family members of common carp confirmed that the sequence we cloned was that of *mc1r* (Figure 2A). Furthermore, this phylogenetic analysis reconstructed the expected taxonomic relationships of the analyzed species adding confidence to the gene identity and conservation of the gene among species.

Protein topology and posttranslational modification sites of *ccMc1rp* were predicted based on its amino acid sequence (Figure 2B). The topology plot also shows the conservation of different parts based on the alignment of amino acid sequences of the common carp and thirteen other fish species. Generally, this protein is very well-conserved across species from different fish orders. However, consistent with its known function, the TM domains are more conserved relative to the extra- and intra-cellular loops and termini.

### Development of black color in Koi larvae

**Figure 3:**
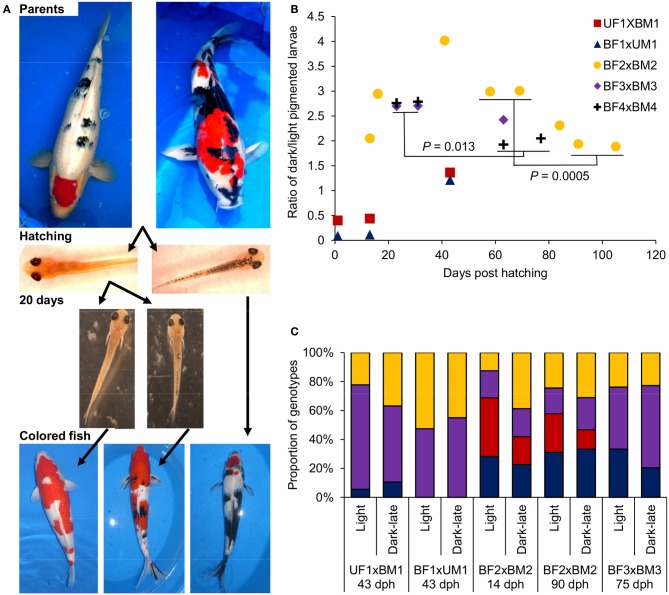
**The development of dark pigmentation in Koi variants and the genotypes of *mc1r* in larvae with and without dark pigmentation. (A)** From top to bottom: images demonstrating the stages of Bekko and Utsuri black patterns development. Crossing a Bekko patterned parent (left) with an Utsuri patterned one (right) yield larvae without (left), and with (right) dark pigmentation already at hatching (i.e., light and dark-early pigmented larvae, respectively). After separation of these light-pigmented larvae and given more time (20 days), a second wave of dark pigmentation development occurs [i.e., light (left) and dark-late (right) pigmented larvae]. Eventually, larvae that remained light-pigmented will become fish without black (left), dark-late pigmented larvae will become fish with the Bekko black pattern (middle) and some of the dark-early pigmented larvae will become fish with the Utsuri black pattern (right). **(B)** Ratio of dark/light pigmented larvae during larval development in five families (Table 2). Two Bekko × Utsuri crosses had variable proportion of dark-pigmented larvae already at hatching. These larvae were separated and thus, the second and third time points of these two families denote the second wave of dark pigmentation. For the three Bekko × Bekko crosses, only one wave of dark-late pigmentation was observed. A statistically significant decrease from 3:1 to 2:1 dark/light ratio was observed in two families. **(C)** *mc1r* microsatellite genotype ratios in light and dark-late pigmented larvae of four families. Differentially colored sections denote the different genotypes in each progeny. The number of genotypes and the expected ratio are different for each family based on the genotypes of the parents (see Table 2) and thus the comparisons between light and dark-late pigmented groups were made only within families. For the BF2 × BM2 family, the genotypic ratios were analyzed at two time points, early and late.

In previous studies, we identified different inheritance modes for the two major Bekko and Utsuri black color patterns in Koi. Here we studied the dynamics of the dark pigmentation development in larvae with respect to these two patterns. Based on our model of black color inheritance in Koi, we have selected parent fish that will enable monitoring the development of the two black color patterns in their progeny (Figure 3A). First, parents with an Utsuri black pattern were crossed to parents with a Bekko black pattern (Table 2) and the development of black pigmentation in larvae of two such progeny groups was monitored. The ratio of dark/light pigmented larvae at hatching was 0.4 for one cross and 0.09 for the other (Figure 3B). Following the groups of light larvae from both families showed that by day 13, a second wave of dark-late pigmentation already started since in one group the dark/light ratio was 0.44 and in the other 0.12 (Figure 3B). This pigmentation wave continued and by day 43, the dark/light ratios in the two families were 1.4 and 1.2 and not different from 1:1 (χ^2^-test, *P* > 0.05). Based on our previous study, some of the larvae with the dark pigmentation at hatching will become fry with Utsuri pattern while larvae that hatched light and developed dark pigmentation only later will become fry with Bekko pattern (David et al., 2004). Therefore, here we found that crossing between parents with the different patterns yielded both patterns in the progeny. The later wave of dark pigmentation development ended with a dark/light ratio not different from 1:1 that fits the expected from a cross between a recessive homozygote (the Utsuri parent) and a heterozygote (the Bekko parent) for a single dominant black color gene.

**Table 2:** **Parent fish and crosses of this study**.

**Female**			**Male**			**Cross label**
**Label**	**Black pattern**	***mc1r* genotype**^**a**^	**Label**	**Black pattern**	***mc1r* genotype**	
UF1	Utsuri	181/202	BM1	Bekko	181/202	UF1 × BM1
BF1	Bekko	202/202	UM1	Utsuri	196/202	BF1 × UM1
BF2	Bekko	181/184	BM2	Bekko	181/202	BF2 × BM2
BF3	Bekko	181/202	BM3	Bekko	181/202	BF3 × BM3
BF4	Bekko	175/202	BM4	Bekko	181/202	BF4 × BM4

a The mc1r microsatellite genotype (allele1/allele2).

Secondly, three pairs of Bekko parents were crossed (Table 2) and the dark pigmentation dynamics was studied in their progeny. In the first few days post hatching, no dark-pigmented larvae were observed in any of the families. In the first family, the dark/light ratio in larvae climbed to 2.1 by day 13 post-hatching, increased to 2.9 by day 16 and further up to 4.0 at day 41 (Figure 3B). This ratio then decreased to 3.0 at days 58 and 69 post-hatching. It could be that the peak of ratio 4.0 observed for this family was a random counting error since such a high ratio was not observed later in this family or in the other families. A similar increase to a dark/light ratio of 2.7 was observed in the other two families at days 23 and 31 (Figure 3B). Therefore, in all three families, the dark/light ratio was not different from 3.0 at some point but the time to reach that point varied among families. Consistent with our previous results, this ratio fits the expected from a cross between two heterozygotes for a single dominant black color gene. However, we continued following the ratio and found that in all three families it dropped down to 2.4–1.9 within a variable number of additional days after which it remained constant (Figure 3B). The final ratios observed in our study for all three families were not significantly different from 2:1. The difference between the earlier higher ratio (around 3:1) and the later lower one (around 2:1) was significant in two of the three families (χ^2^ tests, *P* = 0.0005 and 0.013). This significant decrease in ratio suggests that 1/4 of the overall progeny (or 1/3 of the dark-pigmented progeny) either lost its dark pigmentation or progressively died because of selection at later stages.

### DNA variation in the *mc1r* sequence and Bekko black pattern in Koi

Based on sequencing of the *mc1r* transcript region in genomic DNA of 15 fish, five polymorphisms were identified. One single nucleotide polymorphism (SNP) was found in each of the 5′ and 3′ UTRs and two synonymous SNPs inside the ORF (Figure 1). In addition, we found a microsatellite with an ATT motif located in the 5′ UTR of the gene. Screening 29 individuals from three common carp strains and 63 individuals from four Koi strains revealed six alleles for this microsatellite. The 202, 181 and 184 alleles were the most common accounting together for 94% of the occurrences (Table 3). Sanke are red and white fish with the black Bekko pattern. Among the 44 Sanke fish that we genotyped, 39 had the 202/202, 181/202 or 181/181 genotypes with observed frequencies of 0.570, 0.370 and 0.06, respectively. The observed frequencies match the expected based on Hardey-Weinberg equilibrium (χ^2^-test, *P* = 0.8), suggesting that no selection acted on this locus.

**Table 3:** **Distribution of *mc1r* microsatellite alleles in various carp and Koi strains**.

**Allele size**	**Carp strains**			**Koi strains**				**Total occurrence**
	**Sassan**	**Dor-70**	**Našice**	**Sanke**	**Ohgon**	**Kohaku**	**Utsuri**	
	**(*n* = 10)**^**a**^	**(*n* = 10)**	**(*n* = 9)**	**(*n* = 44)**	**(*n* = 5)**	**(*n* = 3)**	**(*n* = 11)**	
175				2				2
178	1		2	2				5
181	10	9	9	20	2	2	9	61
184	8	11	6	1		1		27
196					2		2	4
202	1		1	63	6	3	11	85
Total occurrence	20	20	18	88	10	6	22	184
Number of alleles	4	2	4	5	3	3	3	6

a For the description of the strain names see materials and methods. Here, Sanke represents the Bekko pattern. In parentheses, n = the number of fish analyzed.

The Koi parents we selected for crosses met two criteria: first, their black color pattern was either Bekko or Utsuri, and second, they were heterozygous in the *mc1r* microsatellite (Table 2). This choice of parents allowed testing for possible linkage between alleles of *mc1r* and the Bekko black pattern. Larvae representing two pigmentation phenotypes, dark-late and light, from each family were sampled. Dark-early pigmentation was not analyzed with respect to *mc1r* alleles since it was absent in this set of families. DNA was extracted from individual larva and the genotype of the *mc1r* microsatellite was determined. The expected ratio of genotypes in each family was determined based on the genotypes of the parents (Table 2) and the observed ratio was measured in all the larvae together and separately within each phenotypic group. For all four families, the distribution of genotypes in all larvae together matched the expected from the parents' genotypes. If linkage between *mc1r* alleles and pigmentation phenotype existed, differences in the ratio of genotypes were expected between the different pigmentation groups. However, we did not find any significant difference in the genotypic ratios between dark-late and light-pigmented larvae in any of the four families (Figure 3C). Consistently, the genotypic ratios in both phenotypic groups were not different from the expected based on the parents' genotypes. Furthermore, the genotypic ratios in dark-late and light groups of family BF2 × BM2 did not change between early (14 dph) and later (90 dph) time points (Figure 3C) although the dark/light phenotypic ratio did change during this time (Figure 3B). These results indicated that sequence variation among *mc1r* alleles does not contribute to the dark-late pigmentation and therefore, to the development of Bekko black pattern in Koi.

### mRNA expression of *mc1r* and dark pigmentation in Koi

**Figure 4:**
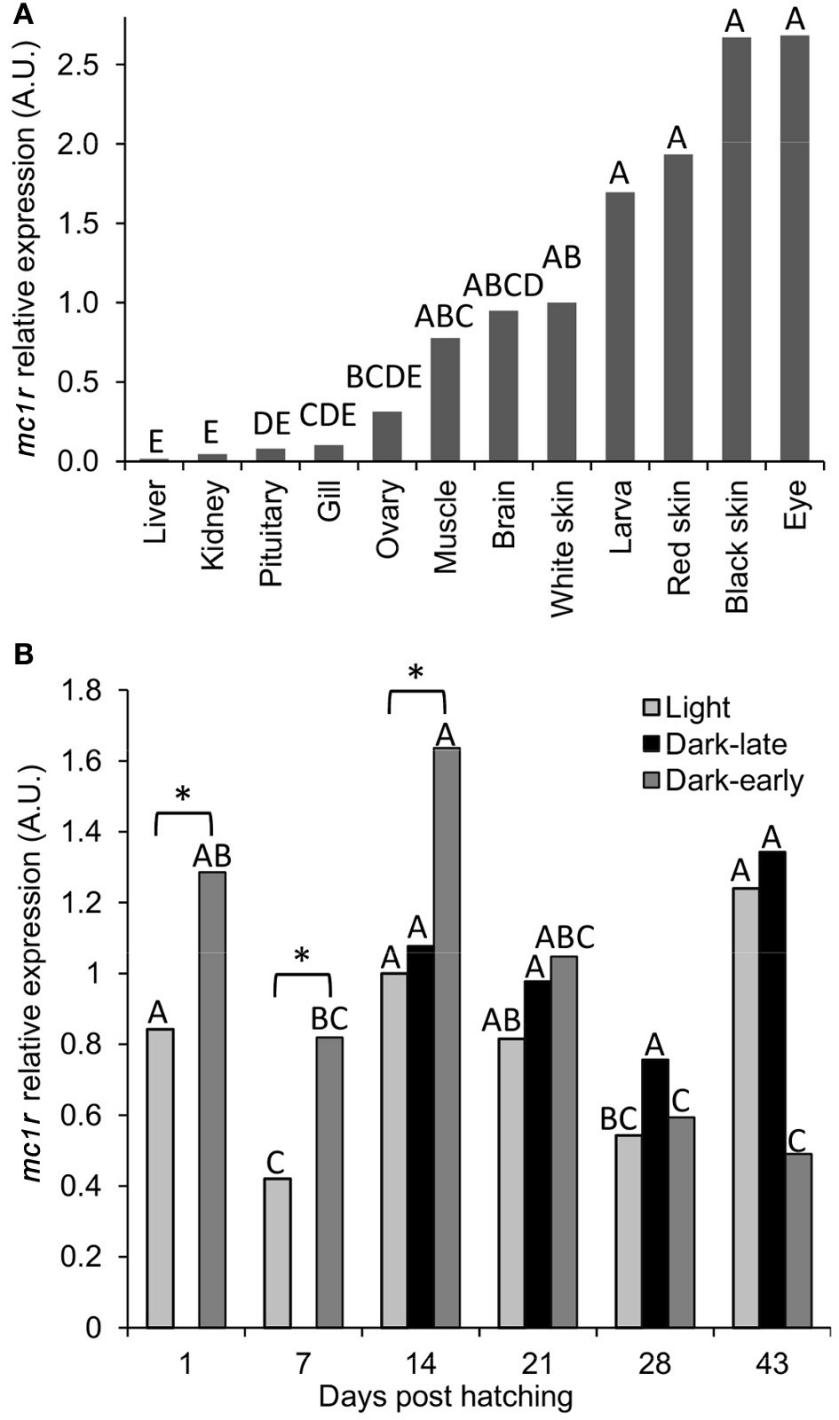
**The expression levels of *mc1r* mRNA in tissues of adult fish and during dark pigmentation development in larvae. (A)** Levels of expression in different tissues were normalized to that of *ef1a* and the fold changes were calculated relative to that in white skin. Bars sharing the same capital letter are not significantly different. **(B)** Levels of expression in light, dark-late and dark-early pigmented larvae at six time-points during development. All measurements were normalized to that of *ef1a* and the fold changes were calculated relative to that in light larvae at day 14. Comparisons were made between time points separately for each phenotypic group and significant differences are denoted by different letters. In addition, marked by asterisk are the only significant (*P* < 0.05) differences between light and dark-pigmented larvae.

The cloning of *ccmc1r* transcript was done from mRNA of dark skin. To further characterize the expression of this gene with respect to pigmentation we measured its mRNA levels in different tissues of adult fish and during development of pigmentation in larvae. The *mc1r* levels were normalized to that of the reference *ef1a* gene. In adult fish, the highest expression was found in black skin and the eye, which is black and thus contains black pigment in it (Figure 4A). Red skin and whole larvae, including their black eyes, had slightly lower expression levels followed by white skin, brain, muscle and ovary. Lowest expression levels were found in gills, pituitary, kidney and liver. The distribution of *mc1r* expression among tissues indicates higher levels in dark-pigmented tissues, compatible with the known involvement of this gene in dark pigmentation. However, considerable expression could be found in other tissues too, suggesting that *mc1r* expression is involved in cellular processes other than pigmentation.

To study the *mc1r* expression levels during the development of dark-pigmentation, dark-early, dark-late and light larvae from the two Utsuri × Bekko families were analyzed at different time-points. The expression level in larvae that hatched dark (dark-early) was higher compared to light larvae, at days 1, 7, and 14 post-hatching (Figure 4B). The expression level in dark-early larvae decreased significantly at days 28 and 43 post-hatching. At day 14, dark-late larvae were already present and *mc1r* expression levels of this group were medium and constant at all of the time points. The expression levels in light larvae increased from day 7 to 14 since at day 14, only about half of the light larvae that will develop dark-late pigmentation already did so (Figure 4B) and analyzed as dark. The other half of the larvae group that would develop dark pigmentation initiated the process but was still phenotypically light and thus, was measured together with light larvae that would remain light throughout the experiment. The expression in light larvae decreased by days 21 and 28, time points at which most of the larvae that would develop dark-late pigmentation already did so and thus, were no longer included in the light larvae group anymore. These measurements indicate that higher expression levels of *mc1r* co-occurred with the development of both early and late dark pigmentation. Taken together, levels of *mc1r* mRNA expression correlated well with dark pigmentation during its development in larvae as well as in tissues of adult fish.

## Discussion

Colors and color patterns are visual cues that serve in inter- and intra-specific communication among animal. Pigmentation defects were also associated with skin diseases such as melanoma cancer. Therefore, much research was devoted to study the genetics and biochemistry of pigmentation [for reviews see (Hoekstra, 2006; Lin and Fisher, 2007; Hofreiter and Schoneberg, 2010; Hubbard et al., 2010; Cieslak et al., 2011)]. Overview of the results, especially those that associate variation in genes with color phenotypes variation, revealed how useful model and domesticated animals were in understanding the genetics of pigmentation also in natural populations (Hubbard et al., 2010; Cieslak et al., 2011). In this work, we continued studying the genetic basis of colors and color development in Koi fish using a candidate gene approach.

In our previous work, we identified the two separate timings of dark pigmentation development in larvae and associated these with the different Bekko and Utsuri black patterns in fry (David et al., 2004). Here, we demonstrated that in larvae of crosses between Utsuri and Bekko parents, these two timings are kept separated and independent from each other, suggesting that the development of these two black patterns are differentially regulated. In addition, we found that the development dynamics of the dark-late pigmentation was variable among families. At day 13 post hatching, the dark/light ratio varied between 0.1 and 2.0. In addition, the time to reach a dark/light ratio of 3.0 and later that of 2.0 was variable among families. Although we repeated the experiments under similar conditions, it could still be that small differences in environmental factors such as temperature, larvae density, and food availability affected significantly the timing and duration of pigmentation development (Parichy et al., 2009). It could also be that genetic variation among families contributed to these developmental differences, since variation existed also between families that were reared in parallel in the same water recirculation system.

The repeated counts of dark/light larvae ratio over time allowed us for the first time to show that ¼ of the fry were selected against and identify the timing of this selection. We prefer explaining the decrease in dark/light ratio by selection rather than by loss of dark pigmentation for two main reasons. First, although our choice of Bekko-patterned parents was phenotypic, neither here nor before (David et al., 2004) did we find any adult parent homozygous for the Bekko-black allele. We have tested so far about 30 parent fish, representing the phenotypic variability in the Bekko patterns, and found no such homozygous adults. The missing ¼ of pigmented fry might be the homozygotes for the black allele. Secondly, for the Utsuri × Bekko crosses, subsets of dark-late larvae were kept separately for mRNA expression analysis. Although only heterozygous for the black allele occurred in these subsets, we did not observe reversion of the pigmentation from dark to light in these groups suggesting that this could not be the reason for the loss of ¼.

Since the gene sequence in the common carp was unknown, we cloned it from cDNA of skin and determined its genomic sequence. Compatible with other studies, we found that the gene sequence of carp as well, was highly conserved compared to other fish species (Selz et al., 2007). Similar to *mc1r* in other fish, the gene in common carp is intronless and probably contained in one copy. Interestingly, the common carp had one additional whole genome duplication compared to the zebrafish and more distantly related species (David et al., 2003), yet this gene returned to a single copy state after that specific duplication. This single copy state in fish and even more so in carp, which experienced a specific duplication, is in contrast to the higher tendency of pigmentation genes to stay duplicated in fish compared to other animals (Braasch et al., 2009).

We selected a candidate gene approach and tested the association between *mc1r* and the monogenic Bekko black pattern in Koi. *mc1r* was an obvious candidate since it was identified as affecting dark pigmentation multiple times in many different animals including fish and often its effect was major, discrete, and dominant (Hoekstra, 2006; Hubbard et al., 2010; Cieslak et al., 2011). Nevertheless, we could not confirm such association in this case. No linkage between *mc1r* genotypes and dark-late pigmentation was found in four families. Furthermore, no significant change in genotypic ratios between dark-late and light larvae was observed because of the selection against 1/3 of the dark-late fry. Finally, also when examining the adult population, the major genotype frequencies of *mc1r* fitted the expected from Hardey-Weinberg equilibrium, although we analyzed only Bekko-patterned fish and although we have failed to find adult homozygotes for the black allele. In studies of pigmentation genes mapping in animals, *MC1R* was over represented relative to other genes (Garcia-Borron et al., 2005; Hofreiter and Schoneberg, 2010). However, the higher number of pigmentation genes and pigmentation cell types in fishes relative to tetrapods suggested different roles for *mc1r* in pigmentation between these taxa (Metz et al., 2006; Richardson et al., 2008; Braasch et al., 2009; Kelsh et al., 2009; Hubbard et al., 2010). Therefore, genes other than *mc1r* might be better candidates for discrete and dominant effects on dark pigmentation in fish. Alternatively, genome wide linkage mapping approach could be useful in identifying the Bekko black color gene in Koi.

The sequence variation of *mc1r* was not associated with the Bekko pattern but the mRNA expression of the gene correlated well with dark pigmentation during development and in adult fish. We found that the expression of the gene increased during times of both dark-early and dark-late pigmentation development in larvae, although different genes probably regulate the two developmental events. This correlation between gene expression and pigmentation development was found despite the variability in the dynamics of the latter among replicate families. Based on this study, measurements of mRNA expression in days of actual development rather than in preset specific days will improve this correlation between the gene's expression level and the development of dark pigmentation. In tissues of adult fish, *mc1r* mRNA expression was higher in pigmented tissues like whole larvae and red skin and even higher in black tissues like black skin and the eye. The expression in tissues of Koi resembled more that in zebrafish than that in platyfish or medaka. While in the zebrafish expression was found in brain, eye, skin and testis, in the other two species it was found in all tissues tested including gills, muscle, ovary, liver, and the above (Selz et al., 2007). We carried out the mRNA measurements using RT-qPCR and obtained more sensitive results than in the other species that were analyzed by a semi-quantitative method. Nevertheless, the differences in tissue expression fit the evolutionary divergence of the species and thus can inform us on the evolution of Mc1r functionality among different fish species.

Taken together, in this study we cloned *mc1r* from the common carp and thus, were able to study its association with black color pattern and development in Koi as well as to measure its expression during development and in adult fish tissues. Identifying the genetic basis of color and color patterns in Koi, will promote the production of this valuable ornamental fish. Furthermore, given the short evolutionary time during which the rich variety of colors and patterns was developed, Koi serves as a good model to unravel pigmentation genes and their phenotypic effects and by that to improve our understanding of the genetic basis of colors also in natural populations.

### Conflict of interest statement

The authors declare that the research was conducted in the absence of any commercial or financial relationships that could be construed as a potential conflict of interest.
